# Numerical Investigation on Drag Reduction Mechanisms of Biomimetic Microstructure Surfaces

**DOI:** 10.3390/biomimetics11010077

**Published:** 2026-01-18

**Authors:** Jiangpeng Liu, Jie Xu, Chaogang Ding, Debin Shan, Bin Guo

**Affiliations:** National Key Laboratory for Precision Hot Processing of Metals, Harbin Institute of Technology, Harbin 150001, China

**Keywords:** drag reduction, biomimetic microstructure, CFD simulation, SST *k*-*ω* model, *Ω* criterion

## Abstract

Biomimetic microstructured surfaces offer a promising passive strategy for drag reduction in marine and aerospace applications. This study employs computational fluid dynamics (CFD) simulations to systematically investigate the drag reduction performance and mechanisms of groove-type microstructures, addressing both geometry selection and dimensional optimization. Three representative geometries (V-groove, blade-groove, and arc-groove) were compared under identical flow conditions (inflow velocity 5 m/s, *Re* = 7.5 × 10^5^) using the shear-stress-transport (SST *k*-*ω*) turbulence model, and the third-generation *Ω* criterion was employed for threshold-independent vortex identification. The results establish a clear performance hierarchy: blade-groove achieves the highest drag reduction rate of 18.2%, followed by the V-groove (16.5%) and arc-groove (14.7%). The analysis reveals that stable near-wall microvortices form dynamic vortex isolation layers that separate the high-speed flow from the groove valleys, with blade grooves generating the strongest and most fully developed vortex structures. A parametric study of blade-groove aspect ratios (*h^+^/s^+^* = 0.35–1.0) further demonstrates that maintaining *h^+^/s^+^* ≥ 0.75 preserves effective vortex-isolation layers, whereas reducing *h^+^/s^+^* below 0.6 causes vortex collapse and performance degradation. These findings establish a comprehensive design framework combining geometry selection (blade-groove > V-groove > arc-groove) with dimensional optimization criteria, providing quantitative guidance for practical biomimetic drag-reducing surfaces.

## 1. Introduction

Drag reduction technologies for marine vessels and other fluid transport systems are of great importance because they can significantly decrease fuel consumption, enhance flow rates, and increase operational efficiency, thereby generating substantial economic and environmental benefits [1,2,3,4]. Over the past decades, a variety of active and passive drag reduction techniques have been proposed, including polymer additives, microbubble injection, compliant coatings, superhydrophobic surfaces, and bioinspired microstructures [5]. Although these approaches have achieved remarkable reductions in frictional drag, many of them still suffer from inherent drawbacks such as high energy consumption, environmental concerns, or limited durability, which restrict their large-scale application.

Natural organisms, through long-term evolution, have developed a rich repertoire of morphological and interfacial strategies to minimize hydrodynamic resistance under harsh environments [6]. Five representative bioinspired concepts have attracted extensive attention in the field of drag reduction: shark skin-inspired microstructure drag reduction, dolphin-skin-inspired compliant walls, penguin-feather-inspired microbubble drag reduction, lotus-leaf-inspired superhydrophobic drag reduction, and pitcher-plant-inspired slippery liquid-infused surfaces [7]. These natural examples demonstrate that appropriately designed surface topography and interfacial properties can passively manipulate the near-wall flow, providing energy-efficient solutions for flow control in marine and fluid engineering.

Among the various bioinspired strategies, interfacial-slip-based approaches such as superhydrophobic surfaces (SHSs), liquid-infused surfaces (LISs), and mucus-inspired slippery coatings have emerged as particularly promising. SHSs combine hydrophobic chemistry with micro/nano-scale roughness to entrap a stable air layer, or plastron, between the roughness elements when submerged [8,9]. The presence of this air–water interface effectively changes the boundary condition from no-slip to partial slip, thereby weakening the near-wall shear and reducing turbulent frictional drag. Extensive reviews and experiments have demonstrated that well-designed SHSs can achieve substantial drag reduction in laminar and turbulent flows, with the drag reduction level strongly depending on texture geometry, gas fraction, and Reynolds number [10]. However, maintaining a stable air layer under high pressure and over long operational periods remains challenging, as plastron depletion and gas dissolution can severely degrade performance.

Inspired by the Nepenthes pitcher plant, slippery liquid-infused porous surfaces (SLIPS) or liquid-infused surfaces (LISs) have been proposed as an alternative to air-based SHSs. In these surfaces, a viscous immiscible lubricant is locked within a textured or porous solid due to capillarity, forming a stable liquid–liquid interface with extremely low contact-angle hysteresis [11,12]. The lubricant layer provides a robust slip boundary, enabling high mobility of aqueous or oily liquids and achieving drag reduction levels on the order of 10–30% for various fluids [13,14]. Compared with SHSs, LISs exhibit better pressure tolerance and self-healing capability; nevertheless, lubricant depletion under continuous shear and mechanical damage is still a critical issue.

Another important class of bioinspired drag-reducing surfaces is based on mucus or mucus-like compliant coatings. Many fish and aquatic organisms secrete a viscoelastic mucus layer on their body surface, which forms a lubricating interface and modifies near-wall turbulence [15]. Experiments have reported drag reduction rates of several tens of percent for fish mucus or mucus-mimicking polymer solutions at appropriate concentrations [16]. Mucus-inspired bionic membranes and slime–groove hybrid surfaces can effectively suppress turbulent fluctuations and attenuate wall-shear stress [17,18]. These mucus or lubricant-based coatings are often combined with underlying microgrooved textures that store and redistribute the liquid phase, again indicating that the geometry of the groove plays a crucial role in determining the overall drag reduction performance. Therefore, a clear understanding of how canonical groove shapes influence the formation of near-wall vortices and local shear stress is essential for the rational design of both hydrophobic and mucus-based drag-reducing coatings.

Despite the rapid development of interfacial-slip technologies, shark-skin-inspired microstructured surfaces remain one of the most attractive strategies for turbulent drag reduction in marine applications, because they are purely passive, require no external energy input, and are compatible with large-scale manufacturing [19,20]. Biological observations reveal that the skin of fast-swimming sharks, such as the shortfin mako shark, is covered with specialized placoid scales whose surfaces exhibit longitudinal riblet-like grooves with characteristic widths below 100 μm [21,22,23,24]. These grooves are aligned with the main flow direction and have been hypothesized to lift and pin streamwise vortices within the viscous sublayer, thereby suppressing lateral momentum transfer and reducing skin-friction drag [19,25,26]. Experimental studies on engineered shark-skin replicas and riblet surfaces—such as triangular, blade-shaped, and semicircular grooves—have demonstrated drag reduction rates of several percent to more than 10% in turbulent flows, confirming the effectiveness of groove-type microstructures [27,28,29,30,31]. “Recent advances in biomimetic riblet design have demonstrated significant potential for practical applications. Mawignon et al. [32] achieved 13.5% drag reduction through optimized cuboidal riblet geometries, while Zani et al. [33] explored multi-scale hierarchical designs for enhanced turbulent boundary layer control. These studies, along with comprehensive reviews by Pakatchian et al. [34], highlight the continued relevance of riblet-based drag reduction research.

Building on these findings, considerable efforts have been devoted to fabricating shark-skin-inspired surfaces using polymers, metals, and composite materials, as well as to exploring their performance in realistic flow conditions. However, most experimental investigations provide limited insight into the internal flow structures inside and above the grooves, because direct measurements of microscale vortices in turbulent boundary layers are extremely challenging. Computational fluid dynamics (CFD) simulations offer a powerful tool to complement experiments by resolving the detailed flow field and elucidating the underlying drag-= reduction mechanisms. Previous numerical studies on riblet and shark-skin-inspired surfaces have revealed that microgrooves can modify the near-wall turbulence structures and reduce wall shear stress [35,36,37]. Nevertheless, many of these works rely on traditional vortex-identification methods such as the *Q* and λ_2_ criteria, which are threshold-dependent and may miss weak vortices close to the wall. Moreover, systematic quantitative relationships between groove geometry, near-wall vortex characteristics, and drag reduction performance have not yet been fully established.

A critical review of the literature reveals three key gaps that currently limit the practical application of riblet-based drag reduction. First, most studies examine single groove geometries without systematic comparison under identical conditions, preventing clear design preferences. Second, while the relationship between groove dimensions and drag reduction has been explored, the effect of aspect ratio (*h^+^/s^+^*) on vortex formation and drag reduction remains poorly understood, lacking quantitative design criteria. Third, previous investigations predominantly rely on traditional vortex identification methods (*Q* criterion, *λ*_2_ method) that require subjective threshold selection, leading to inconsistent vortex identification and hindering quantitative mechanism analysis. The present work addresses these gaps through systematic CFD simulations. Specifically, three representative groove geometries (V-groove, blade-groove, and arc-groove) are systematically compared under identical flow conditions to establish a quantitative ranking of drag reduction performance correlated with vortex metrics. The effect of aspect ratio (*h^+^/s^+^* ranging from 0.35 to 1.0) on drag reduction performance is parametrically investigated using blade-groove microstructures as a representative case to establish optimal design criteria. Furthermore, the third-generation *Ω* vortex-identification criterion is employed for threshold-independent vortex analysis, enabling quantitative correlation between vortex characteristics and drag reduction performance. Together, these investigations provide a comprehensive design framework that bridges the gap between fundamental understanding and practical application of biomimetic drag-reducing surfaces. This work extends the current understanding of biomimetic drag-reducing surfaces beyond traditional *Q* or *λ*_2_ methods.

## 2. Numerical Methodology

### 2.1. Geometry and Model Design

In this study, the drag reduction effects of three types of micro-grooves, including V-groove, blade-groove, and arc-groove, were compared under the same Reynolds numbers. Early research by Walsh at NASA demonstrated that streamwise V-shaped riblets effectively reduce frictional drag when their dimensionless height and spacing satisfy *h*^+^ ≤ 25 and *s*^+^ ≤ 200 [38]. The relationships for these parameters are given by the following equations:

(1) $$s^{+}=0.172\times \frac{sU{Re}^{-0.1}}{v}$$

(2) $$h^{+}=0.172\times \frac{hU{Re}^{-0.1}}{v}$$

where *U* denotes the inlet velocity, *Re* the Reynolds number, and *ν* the kinematic viscosity of the fluid (for water, *ν* = 1 × 10^−6^ m^2^/s) [37].

A representative inflow velocity of 5 m/s was selected for simulation. Because drag reduction occurs primarily under turbulent conditions, the simulations were configured to ensure fully developed turbulence. For flow over a flat plate, turbulence forms when *Re* > 5 × 10^5^.

(3) Re=ρvLµ

In the Reynolds number formula, *L* represents the reference dimension of the boundary layer, which refers to the length of the plate; *μ* is the dynamic viscosity; *ρ* is the fluid density and *v* is the velocity of the incoming flow.

Based on the calculation results, at a flow velocity of 5 m/s, the fluid can reach a turbulent state when the flow direction distance reaches 130 mm. Thus, the streamwise dimension is set to 150 mm, at which the Reynolds number is approximately 7.5 × 10^5^. The ranges of micro-groove heights and widths that meet the drag reduction requirements at an inflow velocity of 5 m/s are shown in Table 1.

**Figure 1:**
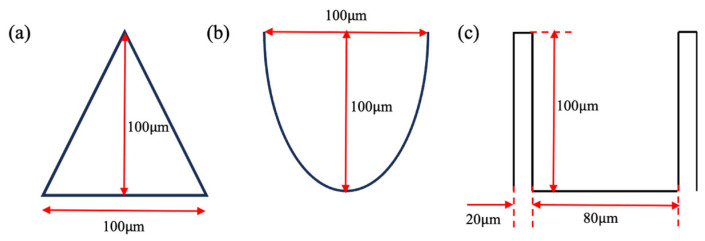
Geometry of microgrooves. (**a**) V-groove, (**b**) arc-groove, and (**c**) blade-groove.

Based on the analysis, an aspect ratio of 1 for the microstructures was selected. Thus, the height and width of the three types of microstructures were all set to 100 μm for the drag reduction analysis, as shown in Figure 1. Both the drag reduction effect and the underlying mechanisms were investigated through numerical simulation.

### 2.2. Establishment of Fluid Drag Reduction Model

Following the above analysis, the streamwise dimension of the model was set to 150 mm. To balance computational efficiency and accuracy, the streamwise dimension of the fluid domain is initially set to 10 mm and then scaled up to 150 mm in Fluent. The analysis focused on the final 10 mm downstream region where turbulence is fully developed. The spanwise dimension was set to ten times the groove width to maintain periodicity.

For turbulent viscous flow, the overall flow can be divided into an outer potential flow region and an inner boundary layer region [39,40]. It is crucial to account for viscous forces inside the boundary layer. The boundary layer thickness is typically much smaller than the characteristic length scale of the flow. However, wall shear stress and other physical quantities near the wall are strongly influenced by viscous effects within the boundary layer [41]. Hence, the boundary-layer thickness *δ* must be considered when defining the domain height. It is estimated using the following:

(4) δ≈0.37×xRex15

where *x* is the streamwise distance and *Re_x_* the local Reynolds number.

**Figure 2:**
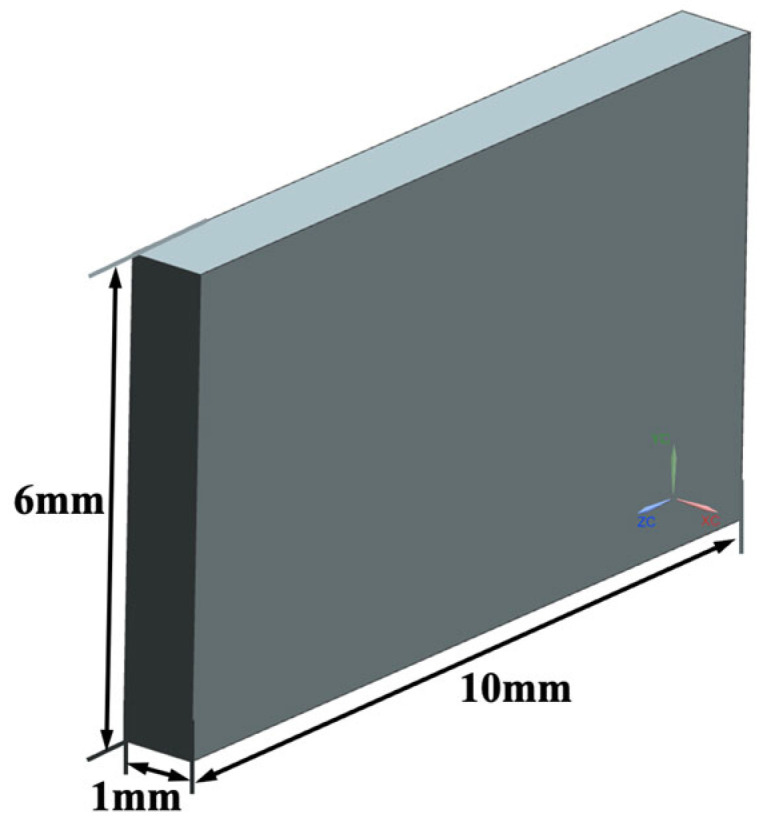
Fluid domain model.

For *Re* = 7.5 × 10^5^, the boundary-layer thickness is approximately 3.7 mm; therefore, the fluid-domain height was set to 6 mm to capture near-wall flow structures. Based on these considerations, the fluid domain model is established as illustrated in Figure 2.

In the mesh generation process, particular attention was paid to the boundary layer formed as the fluid flows along the wall. The fluid velocity gradient is substantial inside the boundary layer, necessitating that the boundary layer mesh maintains good orthogonality with the wall. The first layer height of the mesh is one of the most critical parameters in boundary layer meshing [42]. The dimensionless wall distance *y*^+^ is commonly used to characterize the first mesh layer as follows:

(5) $$y^{+}=\frac{u^{*}y}{v}$$

where u* represents the friction velocity near the wall surface, *y* is the distance between the first layer of mesh nodes and the wall, and *v* is the kinematic viscosity of the fluid.

The formula for calculating the friction velocity near the wall is as follows:

(6) $$u=\sqrt{\frac{\tau_{w}}{\rho }}$$

*τ_w_* represents the wall shear stress, calculated as *τ_w_* = *µ* (*y* = 0, where *μ* is the dynamic viscosity, and *y* is the distance of the first layer of the mesh from the wall).

To further simplify the calculation, the wall friction coefficient, *C_f_*, is introduced, which is defined as follows:

(7) $$C_{f}=\frac{\tau_{w}}{\frac{1}{2}\rho U^{2}}$$

The wall shear stress *τ_w_* can be determined by calculating *C_f_*_._ The calculation of *C_f_* can be performed using Prandtl’s empirical formula when the Reynolds number range is 5 × 10^5^ < *Re* < 10^7^:

(8) $$C_{f}=0.074{Re}^{-\frac{1}{2}}$$

**Figure 3:**
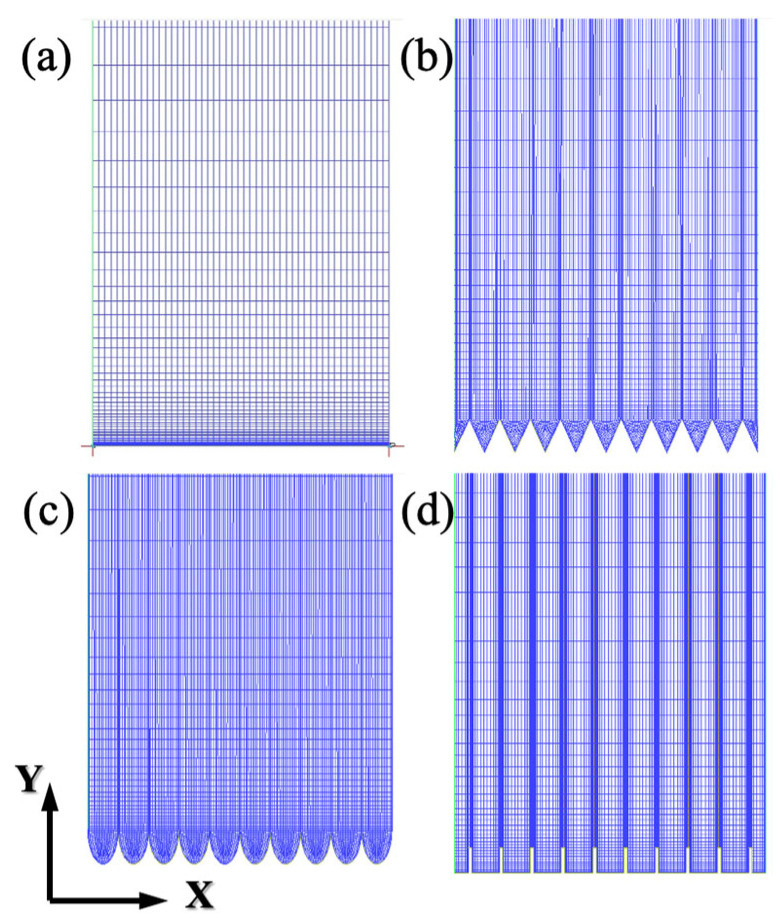
Mesh division of structured grids. (**a**) Smooth surface, (**b**) V-groove, (**c**) arc-groove, and (**d**) blade-groove.

The SST *k*-*ω* model is used for the turbulence model based on previous research. In this case, it is necessary to satisfy *y^+^* ≤1, with a value close to 1 being ideal [43]. Calculations indicate that the first-layer height of the first layer of the mesh should be at least approximately 1.5 μm. The vertical mesh employed an exponential distribution with a ratio set to 1.1, while the streamwise and spanwise directions have a uniform distribution. Mesh generation was performed using ICEM, using structured grids, as illustrated in Figure 3.

### 2.3. Verification of Mesh Independence and Model Accuracy

Increasing the number of mesh cells generally improves the accuracy of numerical simulation calculations, but an excessive number of cells can reduce computational efficiency and increase unnecessary computational costs [44]. To mitigate the impact of mesh density on calculation accuracy and reasonably reduce the required computational power, it is necessary to verify mesh independence. This verification is primarily conducted by comparing the wall friction coefficients at different mesh numbers. Five mesh configurations were tested: 50 × 50 × 50, 50 × 75 × 75, 50 × 100 × 100, 50 × 125 × 125, and 50 × 150 × 150. These mesh configurations are tested for both smooth and microstructure wall models to establish mesh independence.

Mesh independence was first verified for the smooth wall model using five configurations. The SST *k*-*ω* model is selected as the turbulence model, and periodic boundary conditions were applied in the spanwise direction. A velocity inlet boundary condition was specified with a flow speed of 5 m/s, while no-slip conditions were imposed on the walls. A pressure outlet boundary condition was applied. After completing the fluid domain settings, the results are shown in Table 2 and Table 3.

For the smooth wall model, the wall friction coefficient stabilizes around 0.005 when the mesh is set as 50 × 75 × 75. The wall friction coefficient paradoxically increases when the mesh size exceeds 50 × 150 × 150. For microstructure wall models, the simulation results are similar to those of smooth walls. The wall friction coefficient is approximately 0.0012. Therefore, the mesh size is approximately determined to be 50 × 75 × 75 with a total mesh count of about 3 × 10^5^ for subsequent calculations.

To verify the accuracy of SST *k*-*ω* turbulence models in simulating fluid drag reduction, simulations were conducted using a mesh number of 50 × 75 × 75 [45]. The wall friction coefficients obtained from the simulations were compared with empirical values derived from Prandtl’s formula, as shown in Table 4. The results show that the SST *k*-*ω* model keeps the error within 2% of the empirical values, indicating the high accuracy of the SST *k*-*ω* model. Therefore, the SST *k*-*ω* turbulence model will be used in subsequent numerical simulations.

**Figure 4:**
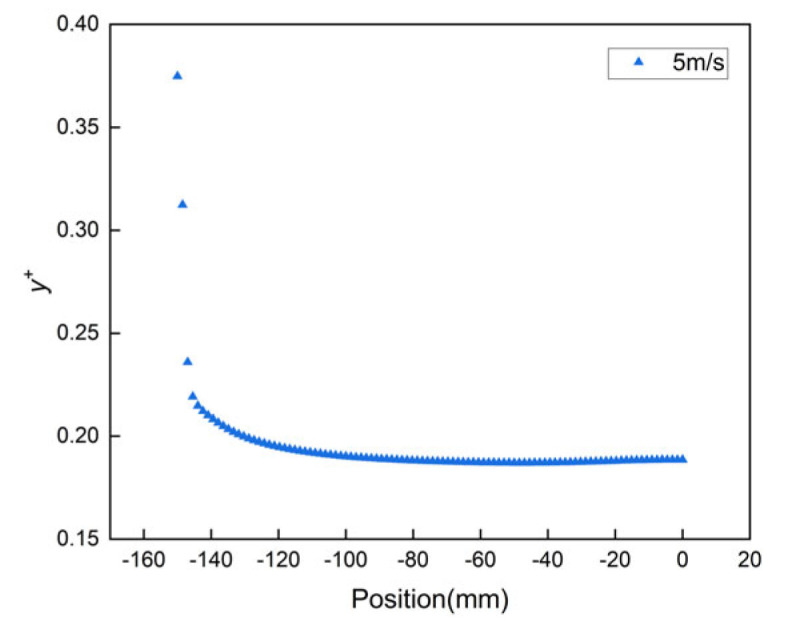
*y^+^* range of the mesh at different velocities.

Considering that the first layer height of the boundary layer mesh was estimated before simulation calculations, the mesh must meet the condition *y^+^* ≤ 1 to ensure the accuracy of the simulation results in the boundary layer since the SST *k*-*ω* turbulence model was used. As shown in Figure 4, the distribution of *y^+^* along the flow direction near the wall shows that after the calculations converge, *y^+^* remains below 1, effectively meeting the requirements of the SST *k*-*ω* model for the *y^+^* range.

## 3. Results and Discussion

### 3.1. Drag Reduction Effect of Microstructures

**Figure 5:**
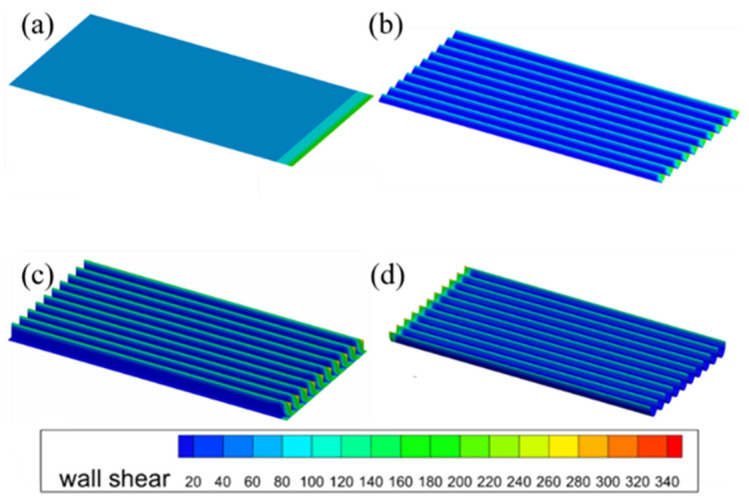
Wall shear stress of different surfaces. (**a**) Smooth surface, (**b**) V-groove, (**c**) blade-groove, and (**d**) arc-groove.

Figure 5 illustrates the wall-shear-stress distributions for the smooth wall and the three microstructured surfaces. The microstructured surfaces significantly affect the distribution and magnitude of the wall shear stress. Excluding the fluid inlet area, the shear stress is uniformly distributed across the smooth surface, with overall high shear stress levels. For V-groove, blade-groove, and arc-groove microstructures, the distribution of shear stress is noticeably uneven, with periodic fluctuations appearing that correspond to the groove geometry. Compared with the smooth wall, the mean wall-shear-stress values on the microstructured surfaces are markedly reduced, confirming the effectiveness of groove-induced drag reduction. The shear stress reaches its peak at the crests of the microstructures and reaches a minimum at the troughs.

**Figure 6:**
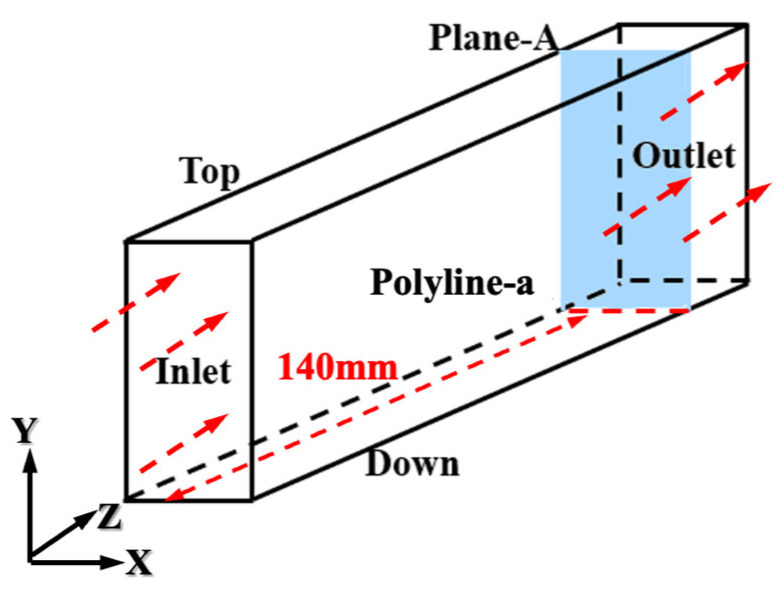
Position of Plane-A and Line-a.

To further investigate the influence of different microstructured surfaces on near-wall flow, a cross-sectional slice, Plane-A, is made at 140 mm from the fluid inlet (where the fluid reaches a full turbulent state). This cross-section intersects with the bottom surface to form a spanwise polyline, Polyline-a, as shown in Figure 6. This approach enables detailed analysis of flow variations across the microstructured region, providing insight into how surface geometry influences near-wall turbulence.

To explore the distribution of wall shear stress affected by different microstructure surfaces further, the shear stress distribution along Polyline-a is extracted using Tecplot. The wall shear stress on microstructured surfaces exhibits periodic variations. The maximum wall shear stress on microstructure surfaces significantly increases compared to that on the smooth surfaces, sometimes by several multiples. However, these stress peaks occur over a very narrow spanwise extent. Consequently, all three microstructure types achieve net reductions in wall shear stress.

**Figure 7:**
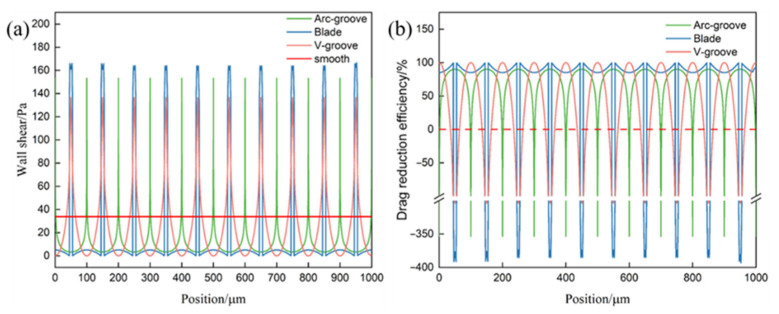
Wall shear stress and drag reduction rate along Polyline-a. (**a**) Distribution of local wall shear stress *τ_w_* along Polyline-a. (**b**) Distribution of the local drag reduction rate *η* along Polyline-a.

The wall shear stress distributions on different microstructured surfaces were analyzed to understand the drag reduction mechanisms. Figure 7a presents the computational domain with the measurement plane indicated, showing the location where wall shear stress data were extracted. The measurement plane is positioned at the mid-span of the microstructured region to capture representative flow characteristics while avoiding edge effects.

To further quantify the drag reduction effects of different microstructure surfaces, a method is proposed that involves comparing the wall shear stress on smooth and microstructure surfaces to characterize the rate of drag reduction [46]. The formula for calculating the drag reduction rate is as follows:

(9) $$\eta =\frac{\tau_{smooth}-\tau_{groove}}{\tau_{smooth}}\times 100\%$$

The drag reduction rate *η* is calculated based on the difference between the wall shear stress of a smooth surface (*τ_smooth_*) and the microstructure surface (*τ_groove_*). When *η* is greater than 0, it exhibits drag reduction; conversely, when *η* is less than 0, it exhibits drag increase.

To quantify the effect of these variations, Figure 7b shows the local drag reduction rate *η* along Polyline-a, calculated from the corresponding wall shear stresses according to Equation (9). Here *η* represents the local reduction of wall shear stress relative to the smooth wall at the same streamwise position. It can be seen that grooved surfaces achieve positive drag reduction over a large portion of the groove period, with pronounced peaks above the groove valleys where the shear stress is strongly suppressed. The blade-groove surface exhibits the highest local drag reduction rate, followed by the V-groove and then the arc-groove, which is consistent with the ranking inferred from the wall shear stress profiles in Figure 7a. In addition, the average drag reduction rate *η* is obtained by calculating along the Polyline-a, where the blade microstructure achieves the highest drag reduction rate, reaching up to 18.2%. The V-groove microstructure follows with a drag reduction rate of 16.5%, and the arc-groove microstructure has a drag reduction rate of 14.7%.

This line-averaged quantity represents the overall reduction of wall shear stress along the evaluation line relative to the smooth wall and follows the same ranking (blade-groove > V-groove > arc-groove) as the local distributions in Figure 7b.

### 3.2. Flow Field and Velocity Distribution

**Figure 8:**
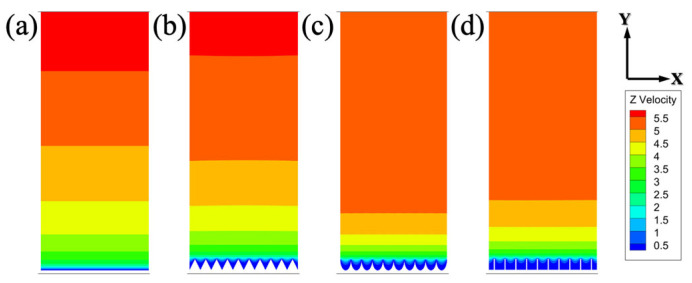
Distribution of fluid velocity in Plane-a of different surfaces. (**a**) Smooth surface, (**b**) V-groove, (**c**) arc-groove, and (**d**) blade-groove.

The influence of different microstructures on the distribution of various physical fields near those surfaces is investigated, as can be seen in Figure 8. The velocity contours over different microstructured surfaces at the cross-section of Plane-A indicate that the velocity gradient is relatively small near the smooth surface, and the area of low velocity near the bottom is much smaller than that near the microstructure surfaces. In addition to having relatively larger low-velocity areas near the microstructure surface, there are also significant velocity gradients apparent near these microstructure surfaces. The phenomenon demonstrates that microstructures on surfaces can significantly affect fluid dynamics, creating regions with varied velocities that can influence the overall behavior of the fluid flow.

To investigate the intrinsic mechanisms of drag reduction by microstructured surfaces further, this paper analyzes the vortex structures near the wall. In previous studies on fluid drag reduction through vortex structure analysis, second-generation criteria such as *Q*, *λ_1_*, *Δ*, and *λ_ci_* are commonly used to identify vortex structures. However, the accuracy of these second-generation criteria depends on threshold values related to specific cases and can be affected by shear, which often influences the determination of vortex structures.

In the present study, the third-generation *Ω* vortex identification method proposed by Liu et al. [47] was adopted for near-wall vortex structure analysis. The *Ω* criterion is defined as the ratio of the vorticity squared to the summation of the vorticity squared and the deformation squared, representing the relative strength of rotation in the flow field. Mathematically, the *Ω* parameter is expressed as follows:

(10) $$\Omega =\frac{{\left\|B\right\|}_{F}^{2}}{{\left\|A\right\|}_{F}^{2}+{\left\|B\right\|}_{F}^{2}+\varepsilon }$$

where ||*A*|| represents the magnitude of the strain rate tensor (symmetric part of the velocity gradient tensor), ||*B*|| denotes the magnitude of the vorticity tensor (antisymmetric part), and ε is a small positive number to avoid division by zero [48]. When *Ω* > 0.5, the rotation dominates over deformation, indicating the presence of a vortex. In practice, *Ω* = 0.52 has been demonstrated to effectively capture vortex structures across various flow conditions without requiring case-specific threshold adjustment.

Compared with traditional vortex identification methods such as the *Q* criterion and the *λ*_2_ method, the *Ω* criterion exhibits several important advantages [48,49,50]. First, it is effectively threshold independent: while *Q* and *λ*_2_ require case-dependent and often subjective selection of iso-values, which can lead to inconsistent vortex identification among different flows, the *Ω* criterion admits a nearly universal threshold (*Ω* = 0.52) that can be applied in a wide range of configurations. Second, *Ω* has a clearer physical interpretation, as it directly quantifies the relative dominance of rotation over strain and is bounded between 0 (pure strain) and 1 (pure rotation), in contrast to the less intuitive magnitudes of *Q* and *λ*_2_ iso-surfaces. Third, the *Ω* method can capture both strong and weak vortices within a single threshold, whereas inappropriate choices of *Q* or *λ*_2_ thresholds may highlight only intense vortices while suppressing weaker but dynamically relevant structures. Finally, *Ω* is a normalized quantity confined to the interval, which facilitates direct and quantitative comparison of vortex structures across different geometries and flow conditions, unlike *Q*, whose magnitude may vary substantially between cases.

To provide quantitative evidence for the vortex-induced drag reduction mechanism, the area-averaged *Ω* values and vortex coverage ratios were calculated for each groove type as shown in Table 5. The blade-groove surface exhibits the highest average *Ω* value of 0.68 in the near-wall region (*y^+^* < 10), compared to 0.61 for V-groove and 0.54 for arc-groove surfaces. Furthermore, the vortex coverage ratio, defined as the percentage of groove area occupied by regions with *Ω* > 0.52, reaches 78% for blade-grooves, 65% for V-grooves, and 52% for arc-grooves. These quantitative metrics directly correlate with the observed drag reduction rates (18.2%, 16.5%, and 14.7%, respectively), providing strong evidence that higher vorticity concentration and greater vortex coverage lead to more effective drag reduction.

**Figure 9:**
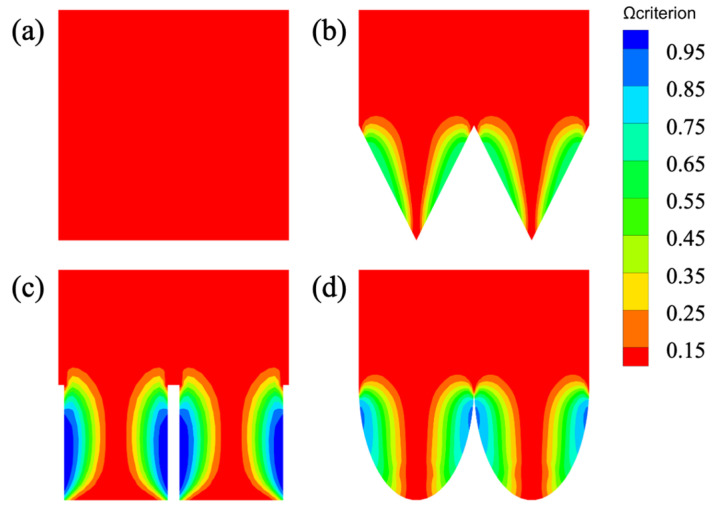
Vortex structures near the wall of different surfaces. (**a**) Smooth surface, (**b**) V-groove, (**c**) blade-groove, and (**d**) arc-groove.

Figure 9 illustrates the near-wall vortex structures identified using the *Ω* criterion across different surface configurations. As expected, the smooth surface (Figure 9a) shows no coherent vortical structures. In contrast, the V-groove surface (Figure 9b) develops symmetric double-vortex patterns within each cavity, with elongated structures extending from the groove tips along both sidewalls; however, the vortex cores do not penetrate to the groove bottom. The blade-groove surface (Figure 9c) displays the most fully developed vortex structures, which occupy the entire cavity depth and form distinct secondary vortex clusters at the groove valleys, thereby creating effective vortex-isolation layers. The arc-groove surface (Figure 9d) exhibits rounded vortex shapes that conform to its curved profile, with penetration depths intermediate between those of V-groove and blade-groove configurations. Notably, the degree of vortex development follows the same ranking as the drag reduction performance: blade-groove > V-groove > arc-groove, confirming the direct link between vortex characteristics and drag reduction effectiveness.

Additionally, combining this with the analysis from the wall shear stress contour maps, it can be observed that for all three types of microstructure surfaces, the areas on either side of the microstructures are regions of low wall shear stress where various sizes of vortex structures are present. These vortex structures effectively separate the microstructure surfaces from the high-velocity fluid regions, preventing direct contact between them and thereby significantly reducing the wall shear stress between the fluid and wall. This mechanism contributes directly to drag reduction. Notably, the blade-groove microstructures exhibit significantly larger vortex structures near the wall compared to the other two types, which aligns with their stronger drag reduction performance as calculated earlier. This correlation confirms the earlier computed drag reduction rate comparisons.

**Figure 10:**
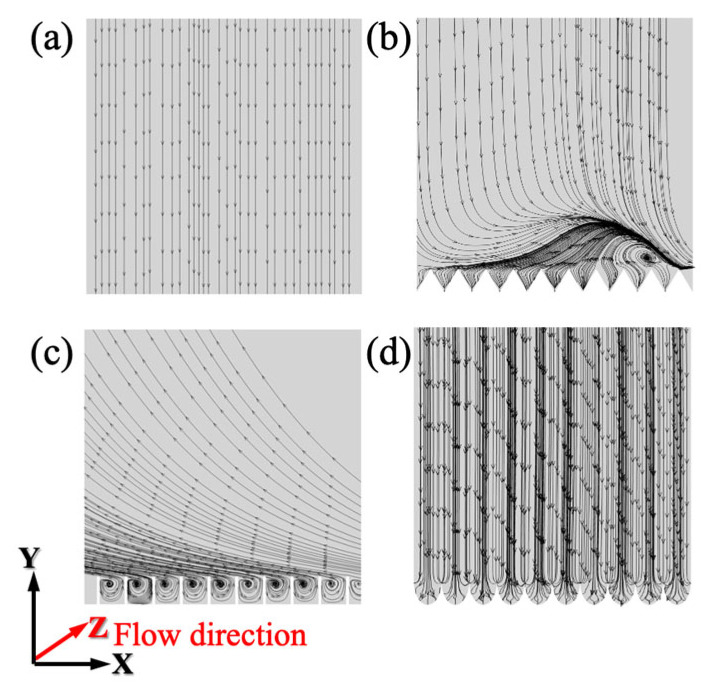
Streamline of different microstructure surfaces in Plane-A. (**a**) Smooth surface, (**b**) V-groove, (**c**) blade-groove, and (**d**) arc-groove.

Based on the streamline distribution diagrams of the smooth and microstructure surface, as shown in Figure 10, small, uniformly distributed vortex clusters form at the bottom of the blade-groove microstructures. The presence of these vortex clusters acts as vortex-isolation layers, effectively reducing fluid drag. Within the V-groove microstructures, complete vortex clusters do not form inside the microstructures. Instead, a larger vortex cluster forms at some distance from the top of the microstructures. Although this also contributes to drag reduction, its effect is weaker than that of the blade-groove microstructures. For the arc-groove microstructures, no significant vortex clusters are observed either inside the microstructures or near the microstructure walls.

### 3.3. Effect of Aspect Ratio on Drag Reduction Performance

To establish practical design guidelines, the influence of groove aspect ratio (*h^+^/s^+^*) on drag reduction performance was systematically investigated using blade-groove microstructures. Five aspect ratios (*h^+^/s^+^* = 1.0, 0.75, 0.6, 0.45, and 0.35) were examined while maintaining a constant groove width of 100 μm. The analysis focused on wall shear stress distribution, velocity gradient, and vortex structure evolution to elucidate the underlying mechanisms.

**Figure 11:**
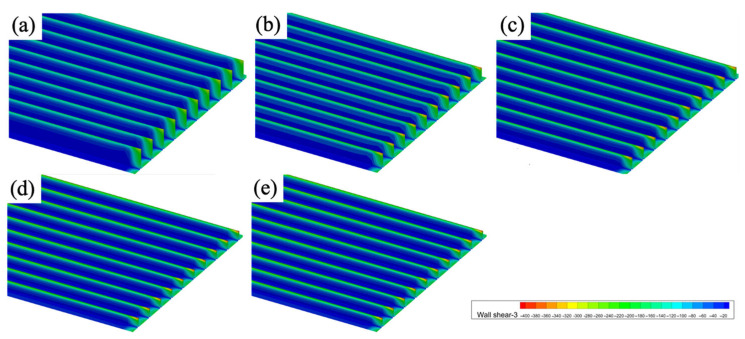
Wall shear stress of different aspect ratios. (**a**) *h^+^/s^+^* = 1.0, (**b**) *h^+^/s^+^* = 0.75, (**c**) *h^+^/s^+^* = 0.6, (**d**) *h^+^/s^+^* = 0.45, and (**e**) *h^+^/s^+^* = 0.35.

Figure 11 presents the wall shear stress distribution for blade-groove surfaces at different aspect ratios. The overall distribution pattern shows high shear stress at the groove tips and low shear stress along the groove sidewalls, which remains consistent across all aspect ratios. However, the aspect ratio significantly affects both the maximum shear stress and the extent of low-stress regions.

**Figure 12:**
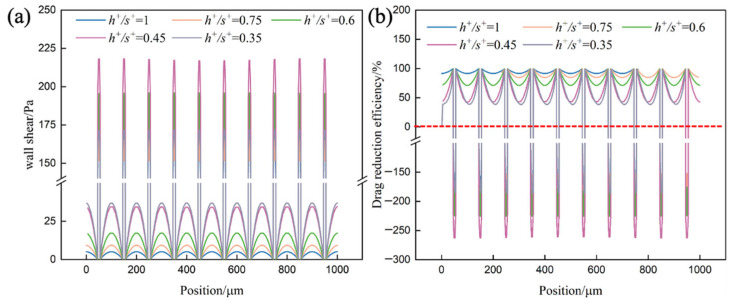
Wall shear stress and drag reduction rate along Polyline-a of different *h^+^/s^+^*. (**a**) Distribution of local wall shear stress *τ_w_* along Polyline-a. (**b**) Distribution of the local drag reduction rate *η* along Polyline-a.

Figure 12 presents the spanwise distributions of wall shear stress (Figure 12a) and local drag reduction rate (Figure 12b) along Polyline-a for different aspect ratios. The wall shear stress profiles reveal a non-monotonic dependence on aspect ratio: peak stress at groove tips increases progressively as *h^+^/s^+^* decreases from 1.0 to 0.45. But then drops at *h^+^/s^+^* = 0.35; meanwhile, stress in the groove valleys rises sharply once *h^+^/s^+^* falls below 0.6. The drag reduction rate profiles directly reflect these stress variations in terms of practical performance. When *h^+^/s^+^* ≥ 0.75, the drag reduction rate remains consistently high across most of the groove period, confirming that effective vortex-isolation layers are maintained. As *h^+^/s^+^* decreases to 0.6 and below, the drag reduction rate in groove valley regions deteriorates markedly, and at *h^+^/s^+^* = 0.35, certain spanwise locations even exhibit negative values, indicating local drag increase rather than reduction. The highest drag reduction rates are achieved at *h^+^/s^+^* = 1.0 and *h^+^/s^+^* = 0.75, demonstrating that higher aspect ratios favor drag reduction. These results collectively establish *h^+^/s^+^* = 0.6 as a critical design threshold, below which drag reduction performance deteriorates rapidly.

**Figure 13:**
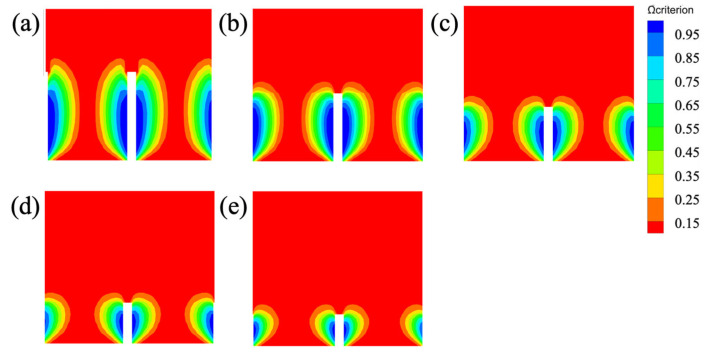
Near-wall vortex structures (identified by *Ω* criterion) for blade-groove surfaces at different aspect ratios. (**a**) *h^+^/s^+^* = 1.0, (**b**) *h^+^/s^+^* = 0.75, (**c**) *h^+^/s^+^* = 0.6, (**d**) *h^+^/s^+^* = 0.45, and (**e**) *h^+^/s^+^* = 0.35.

Figure 13 presents the vortex structures identified by the *Ω* criterion for blade-groove surfaces at different aspect ratios. At *h^+^/s^+^* = 1.0~0.75, well-defined elongated vortex structures form along the groove sidewalls, with distinct secondary vortex clusters occupying the groove valleys. These valley vortices serve as effective vortex-isolation layers that separate the high-speed main flow from the groove bottom. As the aspect ratio decreases to 0.6 and below, the vortex structures undergo significant morphological changes: the elongated sidewall vortices become shorter and wider, and more critically, the secondary vortex clusters in the groove valleys progressively weaken at *h^+^/s^+^* = 0.35. This disappearance of valley vortices directly explains the sharp increase in wall shear stress observed at low aspect ratios, as the protective isolation layer is lost and high-speed flow penetrates deeper into the grooves. Despite these morphological changes, the maximum vorticity magnitude remains relatively constant across all aspect ratios, indicating that the drag reduction degradation at low *h^+^/s^+^* stems primarily from changes in vortex spatial distribution rather than vortex intensity. These observations establish *h^+^/s^+^* = 0.6 as the critical threshold for maintaining effective vortex-isolation layers in blade-groove microstructures.

### 3.4. Engineering Implications

The present study provides quantitative design guidelines for biomimetic drag-reducing surfaces by systematically investigating both groove geometry and dimensional parameters. For the Reynolds number and groove size considered here (groove height and width both 100 μm, aspect ratio *h^+^/s^+^* = 1.0), the blade-groove surface achieves the highest drag reduction rate of 18.2%, followed by the V-groove (16.5%) and arc-groove (14.7%). This clear performance hierarchy demonstrates that groove cross-section geometry significantly influences drag reduction effectiveness, even when overall groove dimensions are constrained by manufacturing considerations.

By combining the distributions of local drag reduction rate along Polyline-a with the near-wall vortex structures identified by the *Ω*-criterion, the analysis reveals that blade-grooves promote a stable and fully developed cavity vortex that effectively isolates the main flow from the groove valleys. For the V-groove surface, the cavity vortex is weaker and high-speed fluid partially penetrates into the grooves, whereas for the arc-groove surface, the flow more easily impinges onto the groove bottom. These differences in vortex topology explain the observed ranking of drag reduction rates among the three geometries.

From an engineering perspective, these findings can be synthesized into practical design guidelines that address both geometry selection and dimensional optimization.

For applications where drag reduction is the primary objective, blade-groove microstructures represent the optimal choice. Their sharp edges generate strong and stable cavity vortices that form effective buffer layers, isolating the high-speed main flow from the wall and substantially reducing wall shear stress. When manufacturing constraints preclude sharp blade edges, V-grooves provide a practical alternative that balances drag reduction performance with geometric robustness and ease of fabrication. Arc-shaped grooves, though less effective for drag reduction, may be preferred in applications where fouling resistance or structural integrity outweighs hydrodynamic performance.

Beyond geometry selection, dimensional optimization is equally critical. The parametric study on blade-groove microstructures demonstrates that maintaining an aspect ratio of *h^+^/s^+^* ≥ 0.75 ensures well-developed vortex-isolation layers and optimal drag reduction, whereas reducing *h^+^/s^+^* below 0.6 triggers vortex collapse and significant performance degradation. These findings establish a comprehensive design framework: the ranking of blade-groove > V-groove > arc-groove guides geometry selection, while the aspect ratio criterion of *h^+^/s^+^* ≥ 0.75 ensures optimal dimensional design for practical biomimetic drag-reducing surfaces.

## 4. Conclusions

This study presents a systematic CFD investigation of biomimetic drag-reducing microstructures, examining groove geometry effects, aspect ratio optimization, and vortex-based drag reduction mechanisms. The principal findings are as follows. A comparative analysis of three representative groove geometries under identical flow conditions (*Re* = 7.5 × 10^5^, groove dimensions 100 μm) reveals a clear performance hierarchy: blade-groove achieves the highest drag reduction rate of 18.2%, followed by V-groove (16.5%) and arc-groove (14.7%). The blade-groove owes its superior performance to sharp edges that induce strong, stable cavity vortices forming effective vortex-isolation layers, which separate the high-speed main flow from the groove valleys and markedly reduce wall shear stress.

A parametric study of blade-groove aspect ratios (*h^+^/s^+^* = 0.35–1.0) demonstrates that dimensional optimization is as important as geometry selection. At *h^+^/s^+^* ≥ 0.75, well-developed vortex-isolation layers persist, and drag reduction remains effective. When *h^+^/s^+^* falls below 0.6, secondary vortex clusters in the groove valleys weaken progressively and eventually vanish, allowing high-speed flow to penetrate deeper into the grooves and causing rapid performance degradation. These results identify *h^+^/s^+^* = 0.6 as a critical design threshold for blade-groove microstructures.

Methodologically, the third-generation *Ω* vortex-identification criterion adopted here provides threshold-independent, quantitative vortex recognition that surpasses traditional *Q* and *λ*_2_ methods. By correlating vortex metrics (average *Ω* values and coverage ratios) directly with drag reduction rates, the *Ω* criterion offers clear mechanistic insight into how groove geometry governs near-wall flow structures.

Collectively, these findings yield a comprehensive design framework for biomimetic drag-reducing surfaces: the geometry ranking (blade-groove > V-groove > arc-groove) guides cross-section selection, while the aspect ratio guideline (*h^+^/s^+^* ≥ 0.75 recommended; *h^+^/s^+^* > 0.6 as the minimum) ensures favorable dimensional design. This framework links fundamental vortex dynamics with practical engineering requirements, offering quantitative guidance for groove-type drag-reducing surfaces in marine and aerospace applications. Future work will pursue experimental validation of optimized blade-groove structures and scalable fabrication methods, such as hot-rolling, to facilitate industrial implementation.

## Figures and Tables

**Table 1:** The range of microstructure heights and widths.

Velocity (m/s)	Reynolds Numbers	Height (µm)	Width (µm)
5	7.5 × 10^5^	≤112	≤900

**Table 2:** Mesh independence of the smooth wall verification scheme.

X × Y × Z	Count of Mesh	*C_f_*
50 × 50 × 50	12,500	0.00498
50 × 75 × 75	281,250	0.00503
50 × 100 × 100	500,000	0.00504
50 × 125 × 125	781,250	0.00505
50 × 150 × 150	1,125,000	0.00510

**Table 3:** Mesh independence of the microstructure wall verification scheme.

X × Y × Z	Count of Mesh	*C_f_*
50 × 50 × 50	12,500	0.00123
50 × 75 × 75	281,250	0.00123
50 × 100 × 100	500,000	0.00153
50 × 125 × 125	781,250	0.00123
50 × 150 × 150	1,125,000	0.00142

**Table 4:** Comparison of simulated values and empirical values of C_f_ incoming.

Flow Velocity (m/s)	Empirical Value of *C_f_*	Simulated Value of *C_f_* (*SST k*-*ω*)	Relative Error (%)
5	0.00495	0.00504	1.8

**Table 5:** Quantitative vortex metrics and drag reduction rates.

Groove Type	Average *Ω*	Coverage %	Drag Red. %
Blade-groove	0.68	78	18.2
V-groove	0.61	65	16.5
Arc-groove	0.54	52	14.7

## Data Availability

The data that support the findings of this study are available from the corresponding author upon reasonable request.

## References

[1] Ahmadzadehtalatapeh M., Mousavi M. (2015). A Review on the Drag Reduction Methods of the Ship Hulls for Improving the Hydrodynamic Performance. Int. J. Marit. Technol..

[2] Bushnell D.M., Moore K.J. (1991). Drag Reduction in Nature. Annu. Rev. Fluid Mech..

[3] Engineering R.A.O. (2013). Future Ship Powering Options: Exploring Alternative Methods of Ship Propulsion.

[4] Gu Y., Yu S., Mou J., Wu D., Zheng S. (2020). Research Progress on the Collaborative Drag Reduction Effect of Polymers and Surfactants. Materials.

[5] Grüneberger R., Kramer F., Wassen E., Hage W., Meyer R., Thiele F., Tropea C., Bleckmann H. (2012). Influence of Wave-Like Riblets on Turbulent Friction Drag. Nature-Inspired Fluid Mechanics: Results of the DFG Priority Programme 1207 “Nature-Inspired Fluid Mechanics” 2006–2012.

[6] Dean B., Bhushan B. (2010). Shark-skin surfaces for fluid-drag reduction in turbulent flow: A review. Philos. Trans. R. Soc. A Math. Phys. Eng. Sci..

[7] Bixler G.D., Bhushan B. (2012). Bioinspired rice leaf and butterfly wing surface structures combining shark skin and lotus effects. Soft Matter.

[8] Lee C., Kim C.-J. (2011). Underwater Restoration and Retention of Gases on Superhydrophobic Surfaces for Drag Reduction. Phys. Rev. Lett..

[9] Samaha M.A., Tafreshi H.V., Gad-El-Hak M. (2012). Superhydrophobic surfaces: From the lotus leaf to the submarine. Comptes Rendus Mécanique.

[10] Lang A., Motta P., Hidalgo P., Westcott M. (2008). Bristled shark skin: A microgeometry for boundary layer control?. Bioinspir. Biomim..

[11] Wong T.-S., Kang S.H., Tang S.K., Smythe E.J., Hatton B.D., Grinthal A., Aizenberg J. (2011). Bioinspired self-repairing slippery surfaces with pressure-stable omniphobicity. Nature.

[12] Epstein A.K., Wong T.-S., Belisle R.A., Boggs E.M., Aizenberg J. (2012). Liquid-infused structured surfaces with exceptional anti-biofouling performance. Proc. Natl. Acad. Sci. USA.

[13] Solomon B.R., Khalil K.S., Varanasi K.K. (2014). Drag reduction using lubricant-impregnated surfaces in viscous laminar flow. Langmuir.

[14] Chen Q.H., Zhang C.Q., Cai Y.K., Luo X., Wang B., Song Q., Liu Z. (2023). Periodically oriented superhydrophobic microstructures prepared by laser ablation-chemical etching process for drag reduction. Appl. Surf. Sci..

[15] Daniel T.L. (1981). Fish mucus: In situ measurements of polymer drag reduction. Biol. Bull..

[16] Hoyt J. (1975). Hydrodynamic drag reduction due to fish slimes. Swim. Fly. Nat..

[17] Oh K.X., Nugroho B., Hutchins N., Monty J.P. Meandering riblets targeting spanwise spatial oscillation of turbulent boundary layers. Proceedings of the 18th Australasian Fluid Mechanics Conference.

[18] Okabayashi K. (2016). Direct numerical simulation for modification of sinusoidal riblets. J. Fluid Sci. Technol..

[19] Walsh M.J. (1983). Riblets as a viscous drag reduction technique. AIAA J..

[20] Bixler G.D., Bhushan B. (2013). Shark skin inspired low-drag microstructured surfaces in closed channel flow. J. Colloid Interface Sci..

[21] Liu Z.-H., Dong W., Xiong Y., Xia F. (2007). Analysis on Factors and Mechanism of Drag Reduction by Grooved Surface. J. Ship Mech..

[22] Lang A.W., Bradshaw M.T., A Smith J., Wheelus J.N., Motta P.J., Habegger M.L., Hueter R.E. (2014). Movable shark scales act as a passive dynamic micro-roughness to control flow separation. Bioinspir. Biomim..

[23] Luo Y., Zhang D., Liu Y. (2015). Recent Drag Reduction Developments Derived from Different Biological Functional Surfaces: A Review. J. Mech. Med. Biol..

[24] Fu Y.F., Yuan C.Q., Bai X.Q. (2017). Marine drag reduction of shark skin inspired riblet surfaces. Biosurface Biotribol..

[25] Luo Y., Liu Y., Zhang D.Y. (2015). Hydrodynamic testing of a biological sharkskin replica manufactured using the vacuum casting method. Surf. Rev. Lett..

[26] Luo Y. (2015). Recent progress in exploring drag reduction mechanism of real sharkskin surface: A review. J. Mech. Med. Biol..

[27] Han X., Zhang D., Li X., Li Y. (2008). Bio-replicated forming of the biomimetic drag-reducing surfaces in large area based on shark skin. Chin. Sci. Bull..

[28] Chen H., Zhang X., Zhang D., Pan J., Hagiwara I. (2013). Large-scale equal-proportional amplification bio-replication of shark skin Based on solvent-swelling PDMS. J. Appl. Polym. Sci..

[29] Graybill M.T., Xu N.W. (2024). Experimental studies of bioinspired shark denticles for drag reduction. Integr. Comp. Biol..

[30] Cui X., Chen D., Chen H. (2023). Multistage gradient bioinspired riblets for synergistic drag reduction and efficient antifouling. ACS Omega.

[31] Mawignon F.J., Qin L., Kouediatouka A.N., Liu J., Djandja O.S., Turay M.C., Winston D.D., Yang H., Wang Z., Wen J. (2025). Optimized three-dimensional cuboidal shark-inspired riblets for enhanced drag reduction in turbulent flow. Ocean Eng..

[32] Mawignon F.J., Liu J., Qin L., Kouediatouka A.N., Ma Z., Lv B., Dong G. (2023). The optimization of biomimetic sharkskin riblet for the adaptation of drag reduction. Ocean Eng..

[33] Zani M.R., Maor N.S., Bhamitipadi Suresh D., Jin Y. (2024). Turbulent boundary layer control with multi-scale riblet design. Energies.

[34] Pakatchian M.R., Rocha J., Li L. (2023). Advances in riblets design. Appl. Sci..

[35] Zhang Y., Ye Z., Li B., Xie L., Zou J., Zheng Y. (2022). Numerical analysis of turbulence characteristics in a flat-plate flow with riblets control. Adv. Aerodyn..

[36] Cafiero G., Iuso G. (2022). Drag reduction in a turbulent boundary layer with sinusoidal riblets. Exp. Therm. Fluid Sci..

[37] Martin S., Bhushan B. (2014). Fluid flow analysis of a shark-inspired microstructure. J. Fluid Mech..

[38] Walsh M.J. Drag characteristics of V-groove and transverse curvature riblets. Proceedings of the Symposium on Viscous Drag Reduction.

[39] Pang R., Sang W., Cai Y. (2019). Drag Characteristics of V-Groove and Transverse Curvature Riblets. Proceedings of the 2018 Asia-Pacific International Symposium on Aerospace Technology (APISAT 2018), Chengdu, China, 16–18 October 2018.

[40] Devenport W.J., Lowe K.T. (2022). Equilibrium and non-equilibrium turbulent boundary layers. Prog. Aerosp. Sci..

[41] Schetz J.A., Bowersox R.D. (2011). Boundary Layer Analysis.

[42] Tsinober A., Bushnell D., Hefner J. (1990). Viscous Drag Reduction in Boundary Layers. Ser Prog. Astronaut. Aeronaut..

[43] Chamorro L.P., Arndt R., Sotiropoulos F. (2013). Drag reduction of large wind turbine blades through riblets: Evaluation of riblet geometry and application strategies. Renew. Energy.

[44] Li C., Tang S., Li Y., Geng Z. (2021). Numerical and experimental investigations on drag-reducing effects of riblets. Eng. Appl. Comput. Fluid Mech..

[45] Yusuf S.N.A., Asako Y., Sidik N.A.C., Mohamed S.B., Japar W.M.A.A. (2020). A short review on rans turbulence models. CFD Lett..

[46] Xu Y., Ruan C., Zhang Z. (2024). Numerical Study on Drag Reduction of Superhydrophobic Surfaces with Conical Microstructures in Laminar Flow. J. Appl. Fluid Mech..

[47] Liu C., Wang Y., Yang Y., Duan Z. (2016). New omega vortex identification method. Sci. China Phys. Mech. Astron..

[48] Dong X.-R., Wang Y.-Q., Chen X.-P., Dong Y., Zhang Y.-N., Liu C. (2018). Determination of epsilon for Omega vortex identification method. J. Hydrodyn..

[49] Wang D.-D., Wang Z.-H., Fan Y.-W., Sun X., Gao Q.-J. (2023). Characterization of vortex structures with self-excited oscillations based on Liutex-Omega vortex identification method. J. Hydrodyn..

[50] Zhang Y.-N., Wang X.-Y., Liu C. (2019). Comparisons and analyses of vortex identification between Omega method and Q criterion. J. Hydrodyn..

